# Global Drinking Water Standards Lack Clear Health-Based Limits for Sodium

**DOI:** 10.3390/nu17132190

**Published:** 2025-06-30

**Authors:** Juliette Crowther, Aliyah Palu, Alicia Dunning, Loretta Weatherall, Wendy Spencer, Devanshi Gala, Damian Maganja, Katrina Kissock, Kathy Trieu, Sera Lewise Young, Ruth McCausland, Greg Leslie, Jacqui Webster

**Affiliations:** 1 The George Institute for Global Health, University of New South Wales, Sydney, NSW 2052, Australia; apalu@georgeinstitute.org.au (A.P.); adunning@georgeinstitute.org.au (A.D.); dmaganja@georgeinstitute.org.au (D.M.); kkissock@georgeinstitute.org.au (K.K.); ktrieu@georgeinstitute.org.au (K.T.); jacqui.webster@uts.edu.au (J.W.); 2 Yuwaya Ngarra-li Partnership, University of New South Wales, Sydney, NSW 2052, Australia; ruth.mccausland@unsw.edu.au; 3 Dharriwaa Elders Group, Walgett, NSW 2832, Australia; loretta@yuwayangarrali.org.au (L.W.); wendy@yuwayangarrali.org.au (W.S.); 4 Walgett Aboriginal Medical Service Limited, Walgett, NSW 2832, Australia; devanshig@walgettams.com.au; 5 Department of Anthropology, Institute for Policy Research, Center for Water Research, Northwestern University, Evanston, IL 60208, USA; sera.young@northwestern.edu; 6 Global Water Institute, University of New South Wales, Sydney, NSW 2052, Australia; g.leslie@unsw.edu.au; 7 Faculty of Health, University of Technology, Sydney, NSW 2007, Australia

**Keywords:** sodium, drinking water, salinity, standards, guidelines, health-based targets, cardiovascular disease

## Abstract

Background/Objectives: High sodium consumption increases the risk of hypertension and cardiovascular disease. Although food remains the primary source of intake, elevated sodium levels in drinking water can further contribute to excessive intake, particularly in populations already exceeding recommendations. This review examines the extent to which national drinking water standards account for sodium-related health risks and aims to inform discussion on the need for enforceable, health-based sodium limits. Methods: National standards for unbottled drinking water in 197 countries were searched for using the WHO 2021 review of drinking water guidelines, the FAOLEX database, and targeted internet and AI searches. For each country, data were extracted for the document name, year, regulatory body, regulation type, sodium limit (if stated), and rationale. Socio-geographic data were sourced from World Bank Open Data. A descriptive analysis was conducted using Microsoft Excel. Results: Standards were identified for 164 countries. Of these, 20% (*n* = 32), representing 30% of the global population, had no sodium limit. Among the 132 countries with a sodium limit, 92% (*n* = 121) adopted the WHO’s palatability-based guideline of 200 mg/L. Upper limits ranged from 50 to 400 mg/L. Only twelve countries (9%) cited health as a rationale. Three countries—Australia, Canada, and the United States—provided a separate recommendation for at-risk populations to consume water with sodium levels below 20 mg/L. Conclusions: Globally, drinking water standards give inadequate attention to sodium’s health risks. Most either lack sodium limits or rely on palatability thresholds that are too high to protect health. Updating national and international standards to reflect current evidence is essential to support sodium reduction efforts. Health-based sodium limits would empower communities to better advocate for safe water. Amid rising water salinity, such reforms must be part of a broader global strategy to ensure universal and equitable access to safe, affordable drinking water as a basic human right.

## 1. Introduction

High sodium intake is a well-established cause of raised blood pressure and a risk factor for cardiovascular disease, contributing to an estimated 1.9 million deaths each year [1]. The World Health Organization (WHO) recommends that adults consume less than 2000 mg of sodium per day [2] but the average global population consumes more than double, at 4300 mg per day [3]. Processed foods and discretionary salt tend to be the highest contributors to sodium intake and, as such, sodium levels in drinking water have not been a focus of sodium reduction efforts [4]. However, drinking water salinity is a growing public health concern and is likely exacerbating health risks already prevalent due to excess sodium consumption. As such, global efforts to reduce sodium intake and to ensure access to safe drinking water must include addressing sodium levels in drinking water.

Most studies investigating the health impacts of sodium have focused on dietary intake without consideration of drinking water sources. However, higher sodium levels in drinking water (greater than 150 mg/L) have been associated with raised systolic and diastolic blood pressure as well as an increased risk of hypertension [5,6,7]. A 2023 scoping review identified 29 studies examining the link between drinking water salinity and blood pressure, with 15 (52%) reporting a positive association [7]. Negative associations reported in 7 (24%) of the studies were mostly attributed to the presence of other minerals, such as magnesium, calcium, and potassium, rather than sodium. Null associations were largely attributed to poor methodology. Most were cross-sectional studies, highlighting the limited longitudinal evidence of associations over time. The strongest associations were observed among younger populations, particularly in randomised trials involving infants [8] and children [9,10]. For example, one prospective study in Massachusetts, USA, comparing two matched cohorts of high-school students exposed to high (272 mg/L) and low (20 mg/L) sodium levels in drinking water, reported a significant increase in systolic and diastolic blood pressure among students in the high-sodium group after controlling dietary sodium intake [11].

More recently, studies in Bangladesh have shown drinking water salinity (>300 mg/L) to be associated in a dose–response manner with an increased risk of preeclampsia, gestational hypertension, and infant mortality [12,13,14]. Additional associations with high drinking water salinity include reduced cognitive performance [15], more frequent hospital visits, and gastrointestinal symptoms including diarrhoea and abdominal pain [16]. Drinking water salinity may also play a role in chronic kidney disease; however, this remains an underexplored area of research [17].

Rising salinity in natural drinking water is an escalating global issue, driven by climate change impacts—such as rising sea levels, more frequent cyclones and storms, and prolonged droughts—along with human activities, like over-extraction of groundwater, alterations to waterways, and industrial and agricultural practices [18]. Sodium levels in drinking water exceeding 250 mg/L have been reported in Brazil [16,19], China [20,21], Italy [22], Kenya [17], United States [23], and Australia [24]. In parts of Bangladesh, sodium levels in drinking water have reached averages of 8210 mg/L during the dry season [5]. The Intergovernmental Panel on Climate Change (IPCC) warns that sea-level rise will likely impact coastal ecosystems in South and Southeast Asia and severely threaten water security in developing countries, with adverse health consequences [18]. Increased water salinity and subsequent health impacts are similarly a concern in drought-stricken regions of Africa [17]. Data remains scarce on how many people are exposed to highsalinity drinking water and the resulting health impacts.

Although salinity in drinking water is increasing and evidence is emerging on the health risks of elevated sodium intake from water, current WHO guidelines and European Union standards legislated in 2023 do not include a health-based limit for sodium. The WHO Guidelines for Drinking Water Quality (GDWQ) have served as an international reference point for the establishment of national and regional water safety standards since 1984. In 2004, the WHO introduced the Framework for Safe Drinking Water [25] to support preventative risk management strategies, including encouraging nations to establish health-based targets to ensure the safety of drinking water, defined as water that ‘does not represent any significant risk to health over a lifetime of consumption’ [26]. Yet, the most recent 2017 GDWQ does not provide a health-based guideline limit for sodium [26]. Instead, a palatability limit for sodium of 200 mg/L is recommended, based on the premise that levels exceeding this ‘may give rise to unacceptable taste’ [26]. The rationale provided by the WHO for not setting a health-based limit is that most sodium intake is dietary and that drinking water typically contributes a small fraction to the total daily intake [4]. However, drinking two litres of water containing the proposed palatability limit would contribute 400 mg of sodium per day, approximately 20% of the upper daily recommended limit for adults. Clearly, the potential contribution of drinking water to daily sodium intake and health impacts may be more significant than currently acknowledged.

The need for more stringent limits on sodium in drinking water is underscored by the experience of Walgett, a regional town in New South Wales, Australia. In 2019, sodium levels in Walgett’s drinking water exceeded 300 mg/L [27]. However, the absence of a health-based limit hindered efforts by local Aboriginal Community Controlled Organisations (ACCOs) to advocate for water quality improvement [27]. These organisations have since called for drinking water guideline updates that reflect the well-established health risks associated with excessive sodium intake [28].

This review, therefore, aims to identify and compare national standards for sodium limits in drinking water globally to provide critical evidence for the need for stronger, unified health-based limits. Amid rising global health concerns about excess sodium intake, along with climate change impacts and human activities driving up drinking water salinity, this research provides support for urgent policy action to protect public health.

### Definitions

*Drinking water standards*: These are national documents detailing the parameters set for drinking water quality. These documents vary in format and include laws, decrees, regulations, standards, specifications, requirements, and guidelines. In this study, we refer to them collectively as standards, acknowledging that the level of enforceability varies depending on both the document type and the country.

*Drinking water guidelines*: These are non-enforceable recommendations provided by health organisations or regulatory agencies, offering guidance on the desirable levels of contaminants in drinking water to maintain water quality. The World Health Organization does not have the authority to enforce standards and, as such, provides global drinking water quality guidelines to support countries in developing their own standards.

*Sodium limit*: This is the maximum sodium concentration in drinking water that is allowed or recommended according to the standards.

*Sodium target*: This is a desirable maximum sodium concentration in drinking water, where a higher concentration is permissible according to the standards.

## 2. Materials and Methods

**Figure 1:**
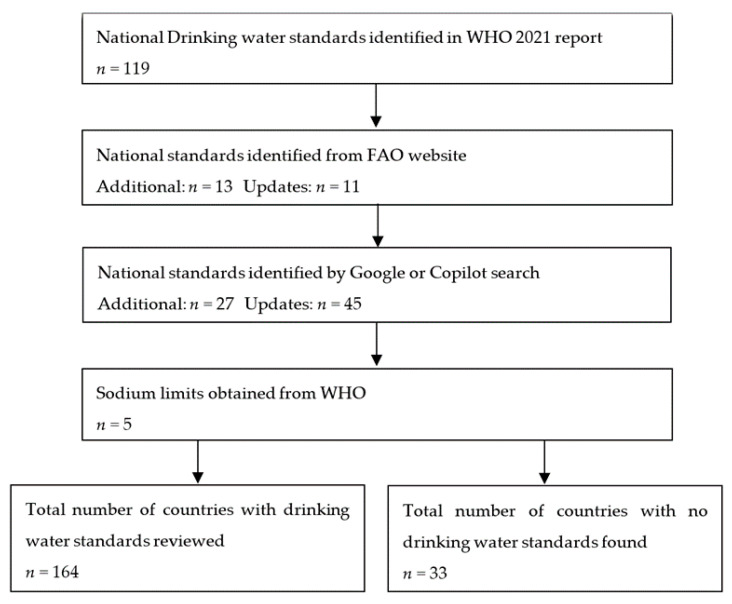
Flowchart of search methods used to identify national drinking water standards for 197 countries.

A desk review of sodium limits in national drinking water standards was conducted from September 2024 to May 2025. National documents for 197 countries were searched for (Figure 1). An internet search using the document title, country, and/or year was conducted to find national water quality documents identified in the 2021 WHO review of national regulations and standards for drinking water quality [25]. The Food and Agriculture Organization (FAO) FAOLEX database—a comprehensive and up-to-date database of national laws, regulations, and policies on food, agriculture, and natural resource management—was used to identify additional and updated national documents [29]. Documents were then searched for using Google and the artificial intelligence (AI) powered software Microsoft Copilot, for example, by asking ‘Is [insert document title and year] the most recent drinking water standard for [insert name of country]?’. For countries where no document could be obtained from searches, national sodium limits identified (but not individually reported) in the 2021 WHO review of national regulations and standards for drinking water were obtained from the staff responsible for the WHO review.

Only standards for unbottled drinking water were included. Standards for commercially sold bottled water, bathing water, surface water, groundwater, or effluent discharge were not included. Documents were included if they were official standards with evidence of what body was responsible for them, and if they outlined specified limits for substances in water. Limits outlined by health or consumer organisations or stipulated in academic material without reference to the source were not included.

National documents written in English were screened by JC and AP. For documents written in Spanish, French, German, Indonesian, Italian, Polish, Portuguese, Russian, and Ukrainian, data were extracted by researchers at The George Institute who were fluent in those languages. For documents written in any other language, Google Translate was used to translate relevant parts of the document. Data extracted included the document title; year; name of the authority responsible for the document; if the standards were mandatory or voluntary; whether the standards included sodium limits and, if so, what those limits were; and, if indicated, if those limits were based on health reasons, aesthetic reasons (organoleptic, palatability, taste, etc.), or another reason.

Socio-demographic data for each country, including population, regional location, gross domestic product (GDP), and income status, were collected using the online World Bank Database (Supplementary Table S1). Data were then analysed, and descriptive statistics were produced using Excel.

## 3. Results

**Figure 2:**
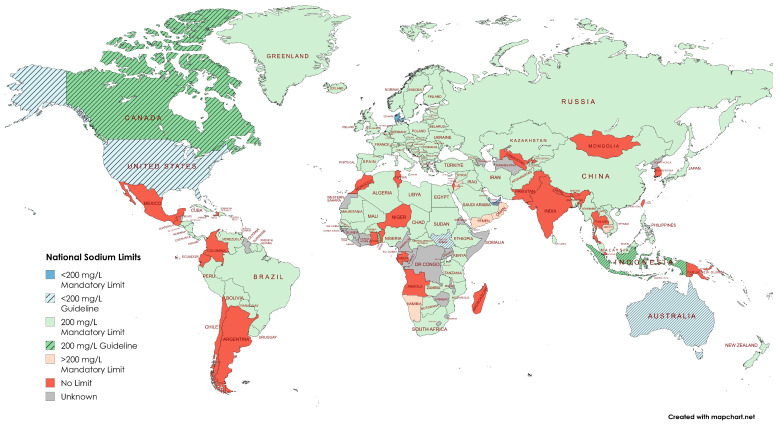
Global map of sodium limits in national drinking water standards.

National documents detailing drinking water quality standards were identified for 164 countries (Table S1). Of these, 132 countries (80%) included specific limits or targets for sodium in drinking water, representing two-thirds (66%) of the global population (Figure 2). In contrast, no sodium limits were specified in the standards of 32 countries (20%), where 30% of the global population resides. No national documents detailing drinking water standards were identified for the remaining 33 countries, which house 5% of the world’s population.

Of the 132 countries that set a sodium limit, the vast majority (n = 121, 92%) set either a target or limit in line with the GDWQ (200 mg/L), with a range from 50 mg/L to 400 mg/L. Six (4%) set a maximum sodium limit higher than the GDWQ, ranging from 250 mg/L to 400 mg/L; however, four of these countries also set a target at or below 200 mg/L. Seven countries (4%) with an upper limit of 200 mg/L also had a guideline target ranging from 25 mg/L to 150 mg/L. Eight countries (5%) set a maximum sodium limit lower than the GDWQ, ranging from 50 mg/L to 180 mg/L.

Only three countries (Australia, Canada, and the United States) provided a recommended limit of 20 mg/L of sodium for people restricting sodium in their diets. The Australian guidelines outlined that people with severe hypertension or congestive heart failure should be aware if the sodium concentration in their drinking water exceeds 20 mg/L. Canada and the United States’ guidelines recommended <20 mg/L of sodium in drinking water for people on strict sodium-reduced diets (<500 mg sodium/day).

**Figure 3:**
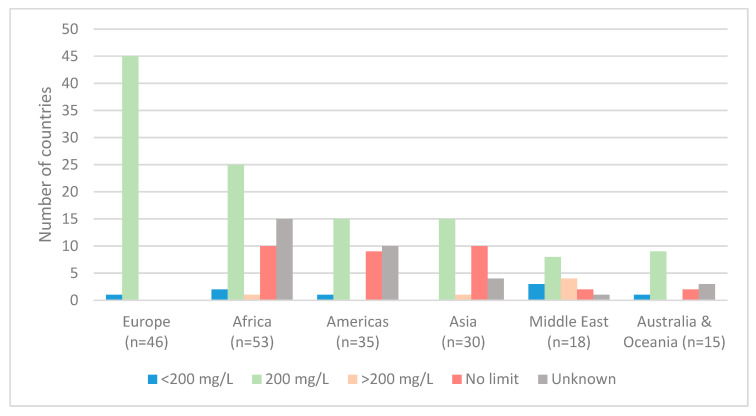
Comparison of sodium limits in national drinking water standards across 197 countries by geographical region.

Comparisons by geographical region demonstrated that nearly all of Europe (98%; n = 45) had a mandatory sodium limit of 200 mg/L, in line with the mandatory 2023 European Union standard [30] (Figure 3). Regions with the highest proportion of countries with no sodium limits were Asia (33%; n = 10) and the Americas (26%; n = 9). The Middle East had the highest number of countries with sodium limits both above 200 mg/L (22%; n = 4) and below 200 mg/L (17%; n = 3). Data were not obtained for a high proportion of countries in Africa (28%; n = 15) and the Americas (29%; n = 10).

**Figure 4:**
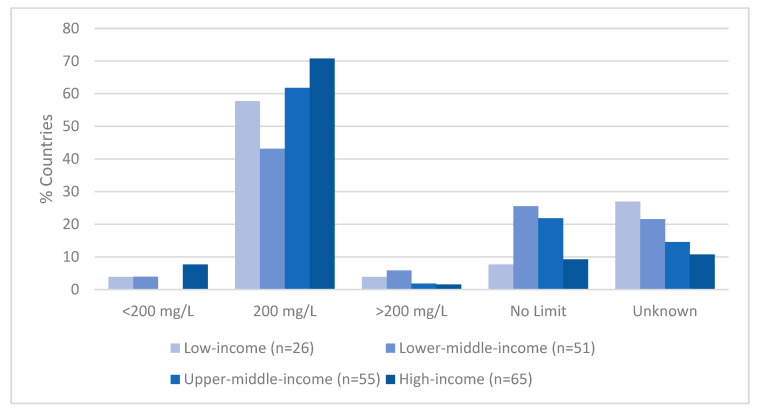
Sodium limits in national drinking water standards by country income level (n = 197).

Among high-income countries, 71% (n = 46) set a sodium limit of 200 mg/L, in line with the WHO guidelines, compared with 43% (n = 22) of lower-middle-income countries (Figure 4). No sodium limits were set for one in four lower-middle-income countries (25%; n = 13) or one in five upper-middle-income countries (22%; n = 12) compared with fewer than one in ten high-income countries (9%; n = 6). Most sodium limits below 200 mg/L were found in high-income countries (63%; n = 5); most limits above 200 mg/L were in lower-middle-income countries (50%; n = 3).

Most (87%; n = 142) of the identified national standards indicated that all limits were mandatory (including sodium, where mentioned). Six national standards (4%) contained non-mandatory limits for sodium in drinking water, including Australia, Canada, Indonesia, Seychelles, Southern Sudan, and Togo. For Cambodia, coastal areas were exempt from having sodium limits. For 15 countries (9%), it was not clear whether sodium limits were mandatory.

In terms of the rationale provided, almost half of the 132 countries with sodium limits (48%; n = 64) described sodium merely as an indicator or physicochemical, inorganic, or similarly termed parameter (Table 1). Over one-quarter of the countries (29%; n = 38) indicated sodium limits were set based on aesthetic reasons (including ‘organoleptic’, ‘taste’, ‘palatability’, ‘sensory’, and ‘consumer acceptability’).

Health and safety reasons for setting upper sodium limits were only referred to in twelve countries’ drinking water standards (9%). Barbados specified a limit of 50 mg/L for sodium, describing it as a chemical that is of health significance in drinking water. Qatar provided a limit of 80 mg/L for sodium in drinking water, citing both health and palatability effects at higher levels. Those recommending an upper limit of 200 mg/L included Bolivia, whose standards indicated that higher values affect health; Russia, Belarus, and Tajikistan, who classified sodium as ‘highly dangerous’ and ‘hazardous’; Costa Rica, Ukraine, and Kyrgyzstan, who listed sodium under ‘Sanitary’ indicators; and Iceland, who indicated that when sodium levels breach recommended limits, the risk to human health should be assessed. Palestine and Namibia had higher sodium limits of 400 mg/L and 300 mg/L, respectively, but cited health, along with taste and infrastructure concerns, for setting these limits. In addition, Ireland’s standards commented that excessive sodium intake can cause hypertension but that the primary mode of intake is via food, and no limit based on health was recommended.

## 4. Discussion

This review highlights the lack of adequate and consistent health-based sodium limits in national drinking water standards globally. One in five countries for which drinking water standards were identified had no sodium limit. Among the 132 countries with a sodium limit, 92% aligned their target or limit with the WHO palatability limit of 200 mg/L [26] and 29% explicitly cited palatability as the reason. Only ten countries were found to explicitly reference health considerations for setting sodium limits. Given the urgent need to reduce population sodium intake to meet Sustainable Development Goal 3.4—reducing premature mortality from non-communicable diseases [3]—the lack of consideration of high water salinity, which in some communities is likely adding to the already excessive sodium intake from food, is a missed public health opportunity.

Low-income countries face the greatest burden from rising drinking water salinity, yet they are the least likely to have sodium limits in place [18]. One in four lower-middle-income countries had no sodium limit, and no standards were available for an additional 27% of low-income countries and 22% of lower-middle-income countries. In the case of Cambodia, sodium limits were even raised from 200 mg/L to 250 mg/L in 2016, with exemptions for coastal areas [31]. Although this likely reflects challenges lower-income countries face in managing rising salinity, it underscores the need for stronger public health protections, not weaker ones. Monitoring and regulating sodium in drinking water is especially critical in high-risk regions, including Southeast Asia.

In contrast, many high-income countries are better equipped to mitigate salinity issues through infrastructure such as desalination [18]. Barbados, for example, enforced the strictest sodium limit in this review at 50 mg/L. They established the Caribbean’s largest brackish water desalination plant in 2000 to address drought-induced water security threats, now supplying water to 30% of its population [32]. Qatar, which relies on desalination for 50% of its water supply, maintains the second strictest limit at 80 mg/L [33]. These examples show that, where infrastructure allows, stricter sodium limits are both feasible and beneficial.

Although lower-income countries should prioritise including sodium limits in their drinking water standards, high-income countries with the capacity to meet lower sodium thresholds should follow the lead of nations like Barbados, Qatar, Sweden, and the Netherlands. The latter two have implemented stricter sodium targets than the 2023 European Union standards of 200 mg/L, anticipating climate-driven salinity risks and aiming to prevent a potential hypertension epidemic [34,35]. These examples highlight the importance of proactive, health-based regulations to protect public health in the face of growing global water quality challenges.

The high number of countries aligning their sodium limits in drinking water standards with WHO guidelines highlights the organisation’s significant influence on national water quality regulation. It is, therefore, concerning that the most recent 2017 WHO GDWQ continues to cite the outdated 2003 Sodium in Drinking-water document as the basis for not establishing health-based targets for sodium [4]. Since that 2003 publication, a substantial body of evidence has been published on the strong links between sodium intake, hypertension, and the risk of cardiovascular disease. Reflecting this, the WHO revised its position in 2012 with the release of its Guideline: Sodium Intake for Adults and Children, which concluded that decreasing sodium intake significantly lowers blood pressure in adults and children and reduces the risk of cardiovascular-related morbidity and mortality [2]. Given that sodium consumed through drinking water contributes to the total daily intake, it would be logical for the WHO to recommend health-based sodium limits in drinking water standards, particularly considering that the communities exposed to high sodium levels in drinking water are often communities already experiencing higher rates of chronic disease [36].

Having separate sodium recommendations for individuals with health conditions is also problematic and inequitable. Three countries, including Australia, recommended lower sodium targets (<20 mg/L) for people who need to reduce their sodium intake for health reasons. However, this recommendation assumes firstly, that health professionals are aware of and will communicate these recommendations, secondly, that information on local water salinity levels is readily available to community members, and finally, that people can access alternative drinking water sources. In communities where the only alternative is to buy expensive bottled water, this has been shown to further exacerbate food insecurity, resulting in increased health inequalities [37]. With one in three adults worldwide suffering from hypertension [38], it would be far more equitable if drinking water standards included enforceable health-based sodium limits.

Furthermore, 200 mg/L of sodium in drinking water is also likely too high for palatability. The level at which sodium can be detected in water varies significantly between individuals and depends on several additional factors, including other chemicals present in the water [39]. A comprehensive investigation by the U.S. Environmental Protection Agency into the taste threshold for sodium in drinking water recommended a limit of 30 to 60 mg/L to ensure palatability for the majority of people [39]. This would also reduce the daily contribution of drinking water to sodium intake to less than 6% of the WHO’s recommended upper limit for sodium.

Drinking water palatability is also closely linked to a range of health-related factors, including food and nutrition insecurity [40]. Unpalatable drinking water can lead to an increased intake of sugary beverages or increased purchasing of bottled water, resulting in reduced expenditure on healthier food items [28]. One study in the United States found that people who did not like the taste of the water or had concerns about the safety of the water switched to soft drinks [41], and a survey in Nicaragua showed that people were switching to bottled water because of the poor taste and health risks of tap water [42]. In a survey among predominantly Aboriginal people in Walgett, Australia, almost half of the respondents reported being water insecure and there was a strong association between water insecurity and food insecurity [43]. Some people in Walgett reported spending as much as fifty Australian dollars a week on bottled water because of poor water quality whilst others reported increased soft-drink consumption [43]. Increased consumption of bottled water and carbonated drinks due to unpalatable drinking water has negative consequences for both population health and the environment [44].

Health-based sodium limits would serve as a powerful advocacy tool for communities like Walgett in Australia facing water quality challenges [45]. Although the Australian Drinking Water Guidelines state that sodium should remain below 180 mg/L [46], this guideline is not mandatory. When sodium levels in the bore water of Walgett exceeded 300 mg/L in 2019, the state health department told residents the water was safe to drink and that if they did not like the taste, they could add mint or low-sugar cordial [47]. Although the government later acknowledged community concerns about the water’s unacceptable palatability due to high sodium levels, a long-term solution has yet to be implemented [45]. Many other regional and remote Australian towns, often with high Aboriginal populations, face similar challenges. Government prioritisation of industrial and agricultural water use, combined with extensive droughts exacerbated by climate change, has left many communities that are reliant on inland rivers with unpalatable, high-sodium groundwater as their primary drinking water source [48]. This situation places already vulnerable populations at greater risk of poor health outcomes, often affecting remote Indigenous communities and compounding existing health inequities [49].

Access to safe drinking water is a basic human right. The United Nations Sustainable Development Goal target 6.1 calls for universal and equitable access to affordable, safe drinking water [50]. Countries significantly vary in their progress toward providing safe water, which is reflected in disparities in national standards [51]. In many low-income nations, the primary challenge lies in establishing basic infrastructure and surveillance systems before sodium levels can be effectively managed [52]. Limited access to potable water can make sodium levels a lower priority. Meanwhile, high-income countries, like Australia, possess the advanced facilities and monitoring frameworks necessary to uphold rigorous water quality standards [53]. For these countries, the focus must shift toward integrating stricter sodium limits into existing regulations and ensuring consistent enforcement to protect public health. Bridging this gap requires global collaboration, resource-sharing, and policy alignment to promote equitable access to safe drinking water for all [53]. The significant influence of WHO guidelines—and, in Europe, the EU standards (which are based on the WHO guidelines)—on national drinking water standards highlights an urgent need for the recommended palatability limit for sodium of 200 mg/L to be revised based on the latest scientific evidence.

This is the first comprehensive review of sodium limits in drinking water standards globally. We used the latest documents publicly available to assess the sodium limits and rationale for those limits as well as the enforceability of national drinking water standards set by countries. The large variation in how different countries set and regulate drinking water standards and how these standards are documented and shared online, along with language barriers, sometimes made it difficult to understand the rationale and/or enforceability of sodium limits in different countries. There are many avenues for future research, including a similar review looking at sodium limits on commercially available bottled water and direct engagement with drinking water agencies to understand how drinking water standards are enforced, whether these limits are being achieved, and what the consequences are for non-compliance. Further research is also required to understand global sodium levels in drinking water and to determine the proportion of people exposed to high-sodium water in each country. Countries vulnerable to climate change impacts would benefit from modelling studies that predict changes in sodium levels to support advocacy for effective mitigation policies. Robust data collection, continuous policy evaluation, and international collaboration are essential for assisting nations in setting and enforcing health-based sodium limits to safeguard public health for future generations.

## 5. Conclusions

There are a lack of adequate and consistent health-based sodium limits in national drinking water standards globally. The vast majority of countries set a sodium limit of 200 mg/L, primarily based on palatability. However, this threshold is problematic because it is too high for health, likely too high for palatability, and falls short of upholding the human right to equitable access to safe and acceptable drinking water. Lower-income nations, along with remote and vulnerable populations in higher-income countries, bear the greatest burden of poor water quality and excessive sodium exposure. With rising water salinity becoming a growing global concern, urgent action is required to address this inequitable health burden. Global recommendations must be updated to reflect the latest scientific evidence and to support populations in meeting WHO sodium intake targets. Amid the growing threat of rising water salinity, reforms to drinking water guidelines and standards must be part of a broader global strategy, led by international public health organisations, to ensure universal and equitable access to safe, affordable drinking water as a fundamental human right.

## Figures and Tables

**Table 1:** A comparison of national criteria on sodium limits in drinking water for 164 countries with the World Health Organization’s 2021 Guidelines for Drinking Water Quality (GDWQ) [25].

Sodium Limit		Country	Rationale
		Angola, Argentina, Armenia, Belize, Benin, Bhutan, Chile, Colombia, Comoros, Ecuador, Gabon, Ghana, Guatemala, Haiti, India, Israel, Madagascar, Marshall Islands, Mauritius, Mexico, Mongolia, Morocco, Nepal, Niger, Pakistan, Papua New Guinea, Singapore, South Korea, Taiwan, Thailand, Tunisia, and Uzbekistan	None
Same as GDWQ (200 mg/L)		Albania, Algeria, Andorra, Austria, Belgium, Bosnia and Herzegovina, Bulgaria, Burundi, Chad, China, Croatia, Cyprus, Djibouti, Dominican Republic, El Salvador, Estonia, Finland, Georgia, Germany, Greece, Hungary, Ireland, Italy (San Marino), Kazakhstan, Kosovo, Kuwait, Latvia, Libya, Liechtenstein, Lithuania, Luxembourg, Malta, Moldova, Monaco, Montenegro, Mozambique, Myanmar, Nigeria, North Macedonia, Panama, Portugal, Romania, Rwanda, Saudi Arabia, Serbia, Seychelles, Slovenia, Spain, Sri Lanka, Sweden, Switzerland, Tanzania, Turkey, Uganda, United Kingdom, Uruguay, and Zambia	Indicator *
		Brazil, Burkina Faso, Central African Republic, Cuba, Czech Republic, Egypt, Ethiopia, Fiji, France, Iraq, Japan, Kenya, Kiribati, Mali, Nauru, New Zealand, Paraguay, Peru, Philippines, Poland, Senegal, Sierra Leone, Slovakia, Solomon Islands, South Africa, Sudan, Syria, Togo, Tonga, Tuvalu, Venezuela, and Vietnam	Palatability ^†^
		Belarus, Bolivia, Iceland, Kyrgyzstan, Russian Federation, Tajikistan, and Ukraine	Health ^‡^
		Afghanistan, Bangladesh, Botswana, Cook Islands, Gambia, Indonesia, Iran, Lao Republic, Malaysia, Maldives, Norway, Samoa, Sao Tome and Principe, and Suriname	Did not specify or unknown
Target (≤GDWQ)	Limit (>GDWQ)		
	250 mg/L	Cambodia (except coastal areas)	Did not specify
100 mg/L	300 mg/L	Namibia	Health/infrastructure
200 mg/L	300 mg/L	Jordan	Palatability
200 mg/L	400 mg/L	Oman	Palatability
200 mg/L	400 mg/L	Palestine	Health/palatability
200 mg/L	400 mg/L	Yemen	Indicator
30–60 mg/L	None	United States of America	Palatability
and 20 mg/L ^§^			
Target (<GDWQ)	Limit (≤GDWQ)		
	50 mg/L	Barbados	Health
	80 mg/L	Qatar	Health/palatability
	100 mg/L	South Sudan	Palatability
	150 mg/L	United Arab Emirates	Indicator
	150 mg/L	Lebanon	Indicator
	150 mg/L	Cabo Verde	Indicator
	175 mg/L	Denmark	Indicator
20 mg/L ^§^	180 mg/L	Australia (guidelines)	Palatability
20 mg/L ^§^	200 mg/L	Canada (guidelines)	Palatability
25 mg/L	200 mg/L	Costa Rica	Health
25 mg/L	200 mg/L	Honduras	Indicator
25 mg/L	200 mg/L	Nicaragua	Indicator
100 mg/L	200 mg/L	Sweden	Indicator
100 mg/L	200 mg/L	Bahrain	Unknown
150 mg/L	200 mg/L	Netherlands	Palatability

* Included terms ‘indicator parameter’, ‘inorganic parameter’, ‘physicochemical parameter’, or similar. No indication was provided as to what the parameter was an indicator for. ^†^ Included terms ‘palatability’, ‘organoleptic’, ‘taste’, ‘aesthetics’, and ‘consumer acceptability’. ^‡^ Included terms ‘affect health’, ‘risk to human health’, ‘dangerous’, ‘hazardous’, and ‘sanitary’. ^§^ Additional recommendation for people on a low-sodium diet (United States and Canada) or with hypertension (Australia). **^¶^** Annual average maximum of 200 mg/L permissible.

## Data Availability

The raw data supporting the conclusions of this article will be made available by the authors on request.

## Supplementary Materials

The following supporting information can be downloaded at https://www.mdpi.com/article/10.3390/nu17132190/s1, Table S1. Data extracted for each country on national drinking water standards and socio-demographics.

## Abbreviations

The following abbreviations are used in this manuscript:

WHO	World Health Organization
GDWQ	Guidelines for Drinking Water Quality
